# Our old friend lithium and encephalopathy: a case report

**DOI:** 10.1192/j.eurpsy.2024.896

**Published:** 2024-08-27

**Authors:** M. P. Cameira, C. F. dos Santos, M. Pereira, M. V. Lisboa, M. R. Soares, P. Abreu

**Affiliations:** ^1^Psiquiatria; ^2^Centro Hospitalar Psiquiátrico de Lisboa, Lisbon, Portugal

## Abstract

**Introduction:**

Lithium is a well-established mood stabilizer used in the management of bipolar disorder, that is generally well-tolerated; however, it is associated with rare but potentially severe neurological side effects. Lithium-induced encephalopathy is characterized by a spectrum of symptoms, ranging from subtle cognitive deficits to severe manifestations such as altered mental status to overt delirium, seizures and coma. Risk factors include advanced age, concomitant medication and underlying renal impairment. This symptoms do not consistentely correlate with lithium concentrations.

**Objectives:**

This abstract aims to provide an overview of the clinical characteristics, underlying mechanisms, and management of lithium-induced encephalopathy.

**Methods:**

We discuss a case of a 62-years-old woman diagnosed with bipolar disorder under treatment with lithium and olanzapine, without recent changes of posology. She presented to emergency department with subacute and fluctuating neuropsychiatric symptoms, including confusion, disorientation in time and space, complex visual hallucinations, delusional ideas, alteration in memory and logic thinking, dysarthria and dyspraxia. Neuroimaging showed no structural abnormalities, blood tests were normal and serum lithium levels were within the therapeutic range (0.8 mEq/L). Upon discontinuation of lithium, the patient exhibited a gradual resolution of symptoms. We conducted a comprehensive search of medical databases, including PubMed, to identify relevant articles related to lithium encephalopathy published up to September 2023.

**Results:**

This case challenges the conventionally established threshold of elevated serum lithium levels in the development of encephalopathy. The underlying pathophysiology is complex and multifactorial, with proposed mechanisms including alterations in neurotransmitter balance, oxidative stress, mitochondrial dysfunction and individual susceptibility to idiosyncratic reactions. Early diagnosis is challenging, necessitating a high clinical suspicion, neuroimaging and exclusion of other etiologies. Management strategies involve discontinuation of lithium, even when serum lithium levels are within the therapeutic range, supportive care, and, in severe cases, hemodialysis to reduce lithium levels rapidly.

**Conclusions:**

Clinicians should maintain a high index of suspicion of lithium-induced encephalopathy, especially in patients presenting with neurological symptoms while on lithium treatment. Early recognition and intervention are essential for minimizing morbidity and preventing potentially irreversible neurological damage. Further research is needed to better understand the precise mechanisms underlying it, risk factors and to refine treatment strategies.

**Disclosure of Interest:**

None Declared

